# We Refer

**Published:** 1874-01

**Authors:** 


We refer with pleasure to the communication of our esteemed friend and talented associate editor, Dr. Harris, of Greensboro, Ga. He "hits the nail upon the head," in relation to the "Red Cross." In that sign we can not conquer the position we aim at for the Record—as the best Medical Journal published in the South. To reach this goal we must have friends and patrons, as practical, devoted, and paying as our respected Greensboro correspondent. We earnestly commend our associate's theory and practice to our friends and readers, with the hope that they will go and do likewise. By a prompt and hearty co-operation our friends will enable us to fill the measure of our aspirations in regard to the excellencies of a journal of which we may modestly, but honestly boast.

